# Crystal structure of [1-(3-chloro­phen­yl)-5-hy­droxy-3-methyl-1*H*-pyrazol-4-yl](*p*-tol­yl)methanone

**DOI:** 10.1107/S2056989015006258

**Published:** 2015-04-02

**Authors:** Balbir Kumar, Kiran J. Nakum, R. N. Jadeja, Rajni Kant, Vivek K. Gupta

**Affiliations:** a Post-Graduate Department of Physics & Electronics, University of Jammu, Jammu Tawi 180 006, India; b Department of Chemistry, Faculty of Science, The M.S. University of Baroda, Vadodara 390 002, India

**Keywords:** crystal structure, 4-acyl­pyrazolone derivative, hydrogen bonding

## Abstract

In the title compound C_18_H_15_ClN_2_O_2_, the dihedral angles between the central pyrazole ring and the pendant chloro­benzene and *p*-tolyl rings are 17.68 (10) and 51.26 (12)°, respectively. An intra­molecular O—H⋯O hydrogen bond is observed, which closes an *S*(6) ring.

## Related literature   

For background to 4-acyl­pyrazolone derivatives, see: Jadeja *et al.* (2012 ▸); Chiba *et al.* (1998 ▸); Marchetti *et al.* (2005 ▸). For related structures, see: Sharma *et al.* (2014 ▸); Abdel-Aziz *et al.* (2012 ▸).
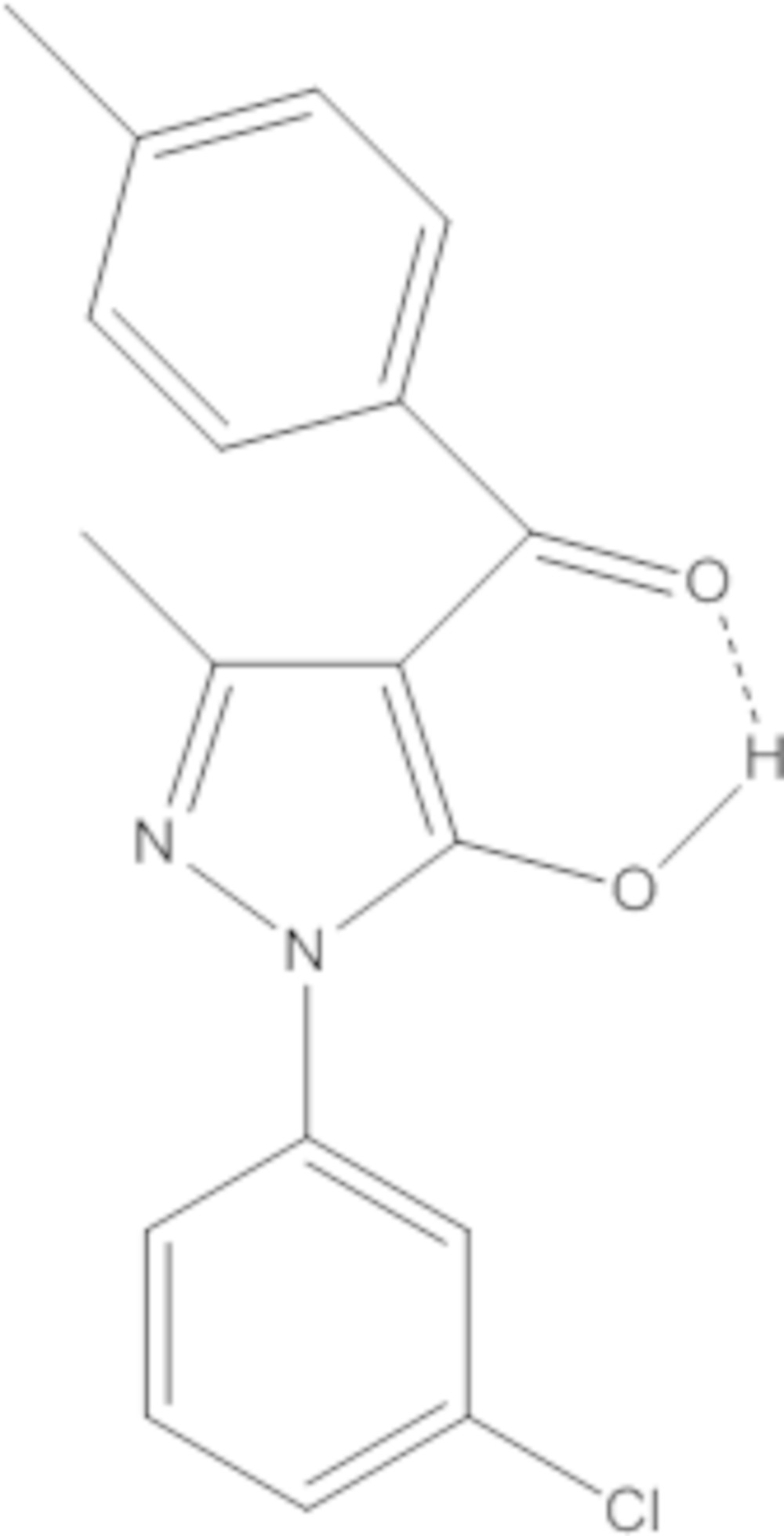


## Experimental   

### Crystal data  

C_18_H_15_ClN_2_O_2_
*M* *_r_* = 326.77
Triclinic, 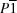

*a* = 5.1469 (5) Å
*b* = 12.0773 (12) Å
*c* = 13.0892 (11) Å
α = 87.247 (7)°
β = 84.396 (7)°
γ = 79.024 (9)°
*V* = 794.57 (13) Å^3^
*Z* = 2
Mo *K*α radiation
μ = 0.25 mm^−1^
*T* = 293 K
0.30 × 0.20 × 0.20 mm

### Data collection  

Oxford Diffraction Xcalibur, Sapphire3 diffractometer
Absorption correction: multi-scan (SCALE3 ABSPACK in *CrysAlis PRO*; Oxford Diffraction, 2010 ▸) *T*_min_ = 0.745, *T*_max_ = 1.000
5633 measured reflections
3099 independent reflections
1411 reflections with *I* > 2σ(*I*)
*R*_int_ = 0.041

### Refinement  

*R*[*F* ^2^ > 2σ(*F* ^2^)] = 0.062
*wR*(*F* ^2^) = 0.164
*S* = 1.00
3099 reflections
210 parameters
H-atom parameters constrained
Δρ_max_ = 0.17 e Å^−3^
Δρ_min_ = −0.20 e Å^−3^

### 

Data collection: *CrysAlis PRO* (Oxford Diffraction, 2010 ▸); cell refinement: *CrysAlis PRO*; data reduction: *CrysAlis PRO*; program(s) used to solve structure: *SHELXS97* (Sheldrick, 2008 ▸); program(s) used to refine structure: *SHELXL97* (Sheldrick, 2008 ▸); molecular graphics: *ORTEP-3 for Windows* (Farrugia, 2012 ▸); software used to prepare material for publication: *PLATON* (Spek, 2009 ▸).

## Supplementary Material

Crystal structure: contains datablock(s) I, New_Global_Publ_Block. DOI: 10.1107/S2056989015006258/hb7373sup1.cif

Structure factors: contains datablock(s) I. DOI: 10.1107/S2056989015006258/hb7373Isup2.hkl

Supporting information file. DOI: 10.1107/S2056989015006258/hb7373Isup3.cml

**Fig. 1.**
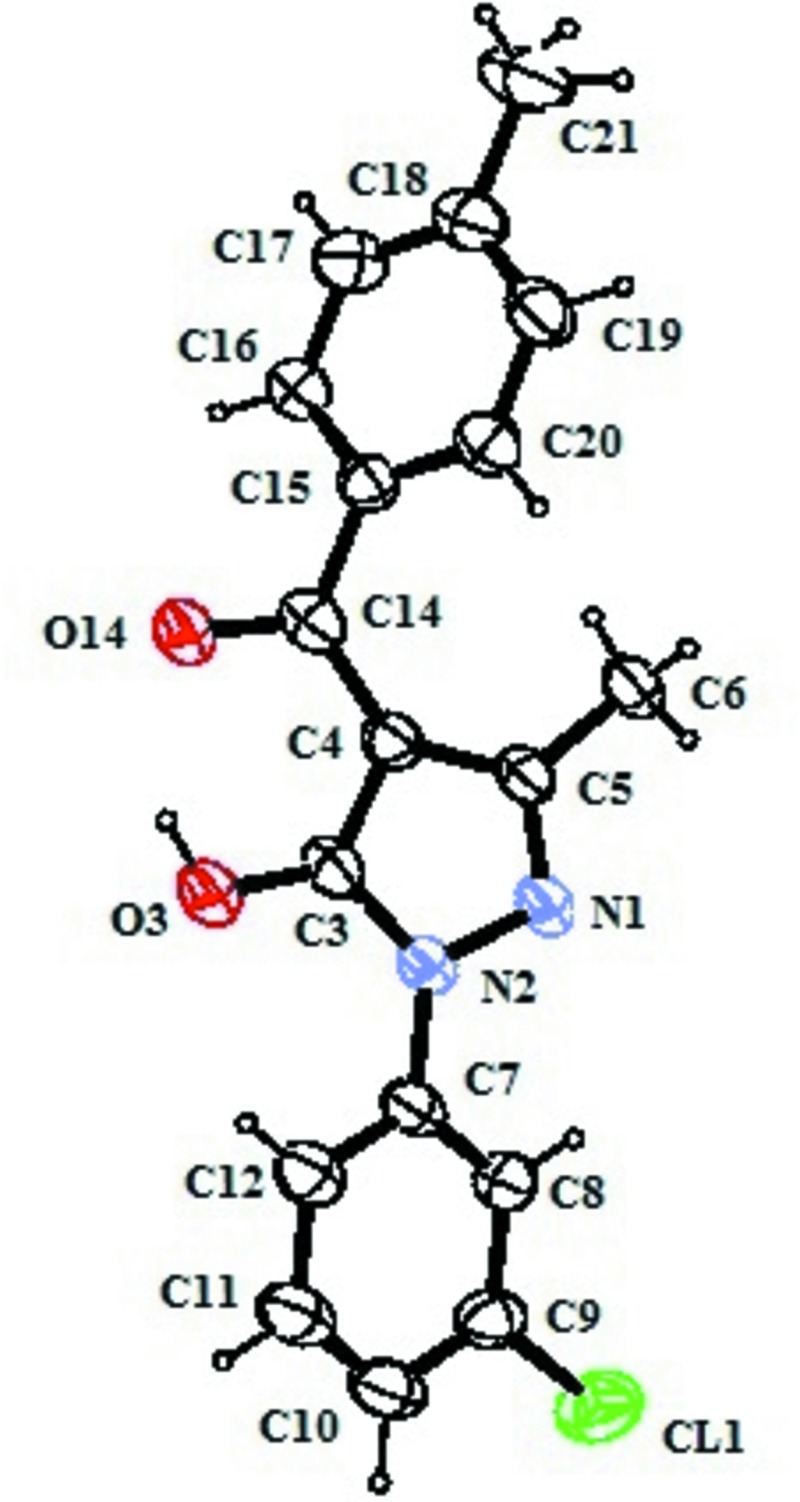
ORTEP view of the title molecule with displacement ellipsoids drawn at the 40% probability level.

**Fig. 2.**
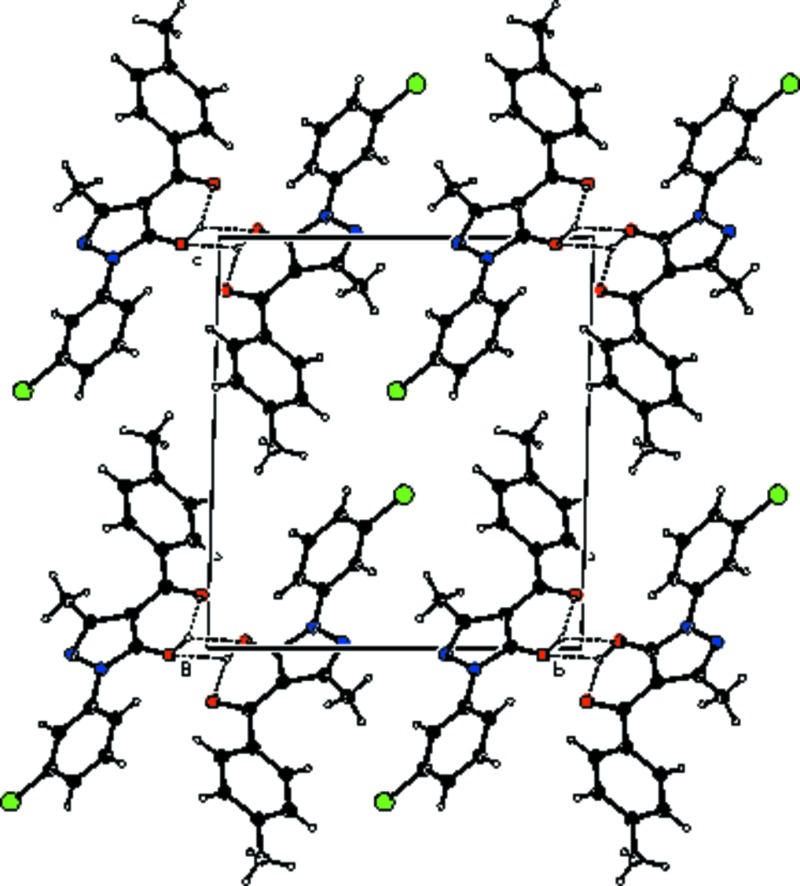
The packing arrangement of molecules viewed down the a axis.

ORTEP . DOI: 10.1107/S2056989015006258/hb7373fig1.tif
*ORTEP* view of the title mol­ecule with displacement ellipsoids drawn at the 40% probability level.

a . DOI: 10.1107/S2056989015006258/hb7373fig2.tif
The packing arrangement of mol­ecules viewed down the *a* axis.

CCDC reference: 1056475

Additional supporting information:  crystallographic information; 3D view; checkCIF report

## Figures and Tables

**Table 1:** Hydrogen-bond geometry (, )

*D*H*A*	*D*H	H*A*	*D* *A*	*D*H*A*
O3H3O14	0.82	1.90	2.581(3)	140

## supplementary crystallographic information

### S1. Comment

4-Acyl-pyrazolones derivatives and their coordination complexes are broadly used in many fields, especially in biological, clinical and analytical applications (Chiba *et al.*, (1998); Marchetti *et al.*, (2005). Due to the presence of two oxygen donor atoms and facile keto-enol tautomerism, they easily coordinate with metal ions after deprotonation of the enolic hydrogen and provide stable metal complexes with six-membered chelate rings. In addition, 4-acyl pyrazolones can form a variety of Schiff bases and are reported to be superior reagents in biological, clinical and analytical applications (Jadeja *et al.*, (2012). In this article we are reporting synthesis and crystal structure of a new 4-acylpyrazolone derivative.

The overall molecular geometry of the title compound, has a normal range and are in good agreement with the corresponding values obtained in case of related structures (Abdel-Aziz *et al.*,2012; Sharma *et al.*, 2014). In the title compound C_18_H_15_Cl_1_N_2_O_2_, all the rings are planar. The dihedral angle between central pyrazole ring and chlorobenzene ring is 17.68 (10)°, between pyrazole ring and *p*-tolyl ring is 51.26 (12)° and between chlorobenzene ring and *p*-tolyl ring is 68.78 (10) °. The bond length of C4- C3 is 1.409 Å that is near to typical C—C double bond indicate that there is double bond between C4—C3. So its geometry becomes planner. The C3—O3 bond(1.291 Å) is much longer than a typical C=O double bond, indicate that C3—O3 bond is single bond and H is attached to O3 and the molecule is in enol form.

### S2. Experimental

1-(3,Chlorophenyl)-3-methyl-5-pyrazolone (20.9 g, 0.1 mol) and 80 ml of dry 1,4-dioxane were placed in a three necked 250 ml round bottom flask equipped with a stirrer, an addition funnel and a reflux condenser. The reaction mass was heated at 70 °C for 10 min. To the resulting yellow solution was added in small portions calcium hydroxide (14.82 g, 0.2 mol) and then toluoyl chloride (15.5 g, 0.1 mol) was added drop wise. During this addition, the whole mass was converted into a thick paste. After the complete addition, the reaction mixture was heated to reflux for 2 h. The yellowish mixture was cooled to room temperature and poured into a 250 ml solution of ice-cold hydrochloric acid (2 *M*) under stirring. The yellow precipitate was filtered, washed with water and dried in a vacuum. After drying a pale-yellow solid was obtained and recrystallized from an acetone-water mixture. (Yield 20.3 g m, 62%). Yellow blocks were obtained by the slow evaporation of the compound in acetone-water mixture (3–4 days).

### S3. Refinement

All the H atoms were geometrically fixed and allowed to ride on their parent Carbon atoms, with C—H distances of 0.93–0.96 Å; and with *U*_iso_(H) = 1.2*U*_eq_(C), except for the methyl groups where *U*_iso_(H) = 1.5*U*_eq_(C),.

### Crystal data

**Table:**

C_18_H_15_ClN_2_O_2_	*Z* = 2
*M**_r_* = 326.77	*F*(000) = 340
Triclinic, *P*1	*D*_x_ = 1.366 Mg m^−^^3^*D*_m_ = 1.37 Mg m^−^^3^*D*_m_ measured by not measured
Hall symbol: -P 1	Mo *K*α radiation, λ = 0.71073 Å
*a* = 5.1469 (5) Å	Cell parameters from 1318 reflections
*b* = 12.0773 (12) Å	θ = 4.1–26.7°
*c* = 13.0892 (11) Å	µ = 0.25 mm^−^^1^
α = 87.247 (7)°	*T* = 293 K
β = 84.396 (7)°	Block, yellow
γ = 79.024 (9)°	0.30 × 0.20 × 0.20 mm
*V* = 794.57 (13) Å^3^	

### Data collection

**Table:**

Oxford Diffraction Xcalibur, Sapphire3 diffractometer	3099 independent reflections
Radiation source: fine-focus sealed tube	1411 reflections with *I* > 2σ(*I*)
Graphite monochromator	*R*_int_ = 0.041
Detector resolution: 16.1049 pixels mm^-1^	θ_max_ = 26.0°, θ_min_ = 3.4°
ω Scan scans	*h* = −6→6
Absorption correction: multi-scan (SCALE3 ABSPACK in *CrysAlis PRO*; Oxford Diffraction, 2010)	*k* = −14→12
*T*_min_ = 0.745, *T*_max_ = 1.000	*l* = −16→15
5633 measured reflections	

### Refinement

**Table:**

Refinement on *F*^2^	Primary atom site location: structure-invariant direct methods
Least-squares matrix: full	Secondary atom site location: difference Fourier map
*R*[*F*^2^ > 2σ(*F*^2^)] = 0.062	Hydrogen site location: inferred from neighbouring sites
*wR*(*F*^2^) = 0.164	H-atom parameters constrained
*S* = 1.00	*w* = 1/[σ^2^(*F*_o_^2^) + (0.0513*P*)^2^] where *P* = (*F*_o_^2^ + 2*F*_c_^2^)/3
3099 reflections	(Δ/σ)_max_ < 0.001
210 parameters	Δρ_max_ = 0.17 e Å^−^^3^
0 restraints	Δρ_min_ = −0.20 e Å^−^^3^

### Special details

**Table:**

Geometry. All e.s.d.'s (except the e.s.d. in the dihedral angle between two l.s. planes) are estimated using the full covariance matrix. The cell e.s.d.'s are taken into account individually in the estimation of e.s.d.'s in distances, angles and torsion angles; correlations between e.s.d.'s in cell parameters are only used when they are defined by crystal symmetry. An approximate (isotropic) treatment of cell e.s.d.'s is used for estimating e.s.d.'s involving l.s. planes.
Refinement. Refinement of *F*^2^ against ALL reflections. The weighted *R*-factor *wR* and goodness of fit *S* are based on *F*^2^, conventional *R*-factors *R* are based on *F*, with *F* set to zero for negative *F*^2^. The threshold expression of *F*^2^ > σ(*F*^2^) is used only for calculating *R*-factors(gt) *etc*. and is not relevant to the choice of reflections for refinement. *R*-factors based on *F*^2^ are statistically about twice as large as those based on *F*, and *R*- factors based on ALL data will be even larger.

### Fractional atomic coordinates and isotropic or equivalent isotropic displacement parameters (Å^2^)

**Table:**

	*x*	*y*	*z*	*U*_iso_*/*U*_eq_	
Cl1	0.3593 (2)	0.51342 (10)	0.37363 (9)	0.1138 (5)	
O14	0.9153 (4)	0.0200 (2)	−0.12991 (19)	0.0789 (8)	
N2	0.6078 (5)	0.2834 (2)	0.0517 (2)	0.0628 (8)	
O3	0.5663 (4)	0.10070 (19)	0.01592 (18)	0.0789 (8)	
H3	0.6322	0.0518	−0.0252	0.118*	
N1	0.7645 (5)	0.3616 (2)	0.0144 (2)	0.0660 (8)	
C7	0.4381 (6)	0.3048 (3)	0.1427 (3)	0.0612 (9)	
C14	0.9791 (6)	0.1164 (3)	−0.1490 (3)	0.0661 (10)	
C8	0.4715 (6)	0.3904 (3)	0.2042 (3)	0.0671 (10)	
H8	0.5998	0.4340	0.1850	0.080*	
C15	1.1641 (6)	0.1285 (3)	−0.2406 (3)	0.0584 (9)	
C4	0.8677 (6)	0.2055 (3)	−0.0821 (3)	0.0596 (9)	
C5	0.9158 (6)	0.3162 (3)	−0.0646 (3)	0.0591 (9)	
C18	1.5098 (7)	0.1494 (4)	−0.4158 (3)	0.0759 (11)	
C16	1.3803 (7)	0.0454 (3)	−0.2639 (3)	0.0721 (10)	
H16	1.4115	−0.0190	−0.2217	0.087*	
C9	0.3107 (7)	0.4095 (3)	0.2944 (3)	0.0751 (11)	
C11	0.0854 (7)	0.2626 (4)	0.2618 (3)	0.0858 (12)	
H11	−0.0425	0.2188	0.2814	0.103*	
C20	1.1174 (7)	0.2212 (3)	−0.3063 (3)	0.0737 (10)	
H20	0.9691	0.2774	−0.2921	0.088*	
C3	0.6724 (6)	0.1890 (3)	−0.0040 (3)	0.0626 (9)	
C12	0.2420 (6)	0.2418 (3)	0.1708 (3)	0.0733 (11)	
H12	0.2166	0.1859	0.1284	0.088*	
C6	1.1176 (6)	0.3801 (3)	−0.1179 (3)	0.0733 (11)	
H6A	1.0483	0.4191	−0.1780	0.110*	
H6B	1.2784	0.3282	−0.1377	0.110*	
H6C	1.1544	0.4338	−0.0721	0.110*	
C10	0.1147 (7)	0.3465 (4)	0.3240 (3)	0.0851 (13)	
H10	0.0058	0.3612	0.3847	0.102*	
C17	1.5527 (7)	0.0571 (4)	−0.3503 (3)	0.0835 (12)	
H17	1.7012	0.0009	−0.3641	0.100*	
C19	1.2894 (8)	0.2317 (4)	−0.3935 (3)	0.0827 (12)	
H19	1.2552	0.2949	−0.4370	0.099*	
C21	1.6955 (8)	0.1619 (4)	−0.5112 (3)	0.1139 (16)	
H21A	1.6488	0.1217	−0.5661	0.171*	
H21B	1.8752	0.1316	−0.4970	0.171*	
H21C	1.6802	0.2404	−0.5308	0.171*	

### Atomic displacement parameters (Å^2^)

**Table:**

	*U*^11^	*U*^22^	*U*^33^	*U*^12^	*U*^13^	*U*^23^
Cl1	0.1352 (10)	0.1143 (10)	0.0849 (9)	−0.0078 (7)	0.0057 (7)	−0.0276 (8)
O14	0.0959 (17)	0.0706 (17)	0.0744 (18)	−0.0365 (14)	0.0131 (13)	−0.0027 (14)
N2	0.0660 (17)	0.0642 (19)	0.0591 (19)	−0.0223 (14)	0.0078 (14)	0.0016 (16)
O3	0.0893 (16)	0.0740 (17)	0.0770 (19)	−0.0360 (14)	0.0159 (14)	−0.0026 (15)
N1	0.0724 (17)	0.064 (2)	0.063 (2)	−0.0256 (15)	0.0060 (15)	0.0046 (15)
C7	0.0538 (19)	0.071 (2)	0.053 (2)	−0.0069 (17)	0.0046 (16)	0.0139 (18)
C14	0.068 (2)	0.079 (3)	0.054 (2)	−0.0241 (19)	−0.0048 (17)	0.008 (2)
C8	0.070 (2)	0.065 (2)	0.062 (2)	−0.0086 (18)	0.0025 (18)	0.0011 (19)
C15	0.069 (2)	0.061 (2)	0.049 (2)	−0.0225 (17)	−0.0024 (17)	−0.0053 (18)
C4	0.0609 (19)	0.065 (2)	0.056 (2)	−0.0222 (17)	0.0037 (16)	−0.0032 (18)
C5	0.065 (2)	0.063 (2)	0.050 (2)	−0.0180 (17)	0.0011 (16)	0.0082 (17)
C18	0.078 (2)	0.103 (3)	0.056 (3)	−0.041 (2)	0.000 (2)	−0.008 (2)
C16	0.083 (2)	0.070 (3)	0.061 (3)	−0.015 (2)	0.0024 (19)	0.006 (2)
C9	0.085 (2)	0.080 (3)	0.050 (2)	0.007 (2)	−0.0008 (19)	0.002 (2)
C11	0.071 (2)	0.104 (3)	0.078 (3)	−0.017 (2)	0.014 (2)	0.008 (3)
C20	0.089 (2)	0.075 (3)	0.056 (3)	−0.014 (2)	−0.0051 (19)	0.006 (2)
C3	0.064 (2)	0.070 (2)	0.058 (2)	−0.0267 (18)	0.0003 (17)	0.0068 (19)
C12	0.062 (2)	0.084 (3)	0.072 (3)	−0.0156 (19)	0.0028 (19)	0.007 (2)
C6	0.086 (2)	0.072 (3)	0.065 (2)	−0.0315 (19)	0.0070 (19)	0.007 (2)
C10	0.078 (3)	0.100 (3)	0.064 (3)	0.002 (2)	0.016 (2)	0.018 (2)
C17	0.078 (2)	0.095 (3)	0.073 (3)	−0.011 (2)	0.006 (2)	−0.007 (3)
C19	0.106 (3)	0.088 (3)	0.059 (3)	−0.035 (2)	−0.006 (2)	0.012 (2)
C21	0.112 (3)	0.170 (5)	0.071 (3)	−0.069 (3)	0.024 (2)	−0.011 (3)

### Geometric parameters (Å, º)

**Table:**

Cl1—C9	1.735 (4)	C18—C19	1.376 (5)
O14—C14	1.276 (4)	C18—C21	1.516 (5)
N2—C3	1.352 (4)	C16—C17	1.386 (4)
N2—N1	1.397 (3)	C16—H16	0.9300
N2—C7	1.410 (4)	C9—C10	1.390 (5)
O3—C3	1.293 (3)	C11—C10	1.369 (5)
O3—H3	0.8200	C11—C12	1.377 (4)
N1—C5	1.307 (4)	C11—H11	0.9300
C7—C8	1.386 (4)	C20—C19	1.391 (4)
C7—C12	1.388 (4)	C20—H20	0.9300
C14—C4	1.418 (4)	C12—H12	0.9300
C14—C15	1.477 (4)	C6—H6A	0.9600
C8—C9	1.376 (4)	C6—H6B	0.9600
C8—H8	0.9300	C6—H6C	0.9600
C15—C16	1.370 (4)	C10—H10	0.9300
C15—C20	1.376 (5)	C17—H17	0.9300
C4—C3	1.396 (4)	C19—H19	0.9300
C4—C5	1.438 (4)	C21—H21A	0.9600
C5—C6	1.508 (4)	C21—H21B	0.9600
C18—C17	1.371 (5)	C21—H21C	0.9600
			
C3—N2—N1	110.0 (2)	C10—C11—H11	119.4
C3—N2—C7	129.8 (3)	C12—C11—H11	119.4
N1—N2—C7	119.7 (3)	C15—C20—C19	120.8 (3)
C3—O3—H3	109.5	C15—C20—H20	119.6
C5—N1—N2	106.5 (2)	C19—C20—H20	119.6
C8—C7—C12	120.3 (3)	O3—C3—N2	123.5 (3)
C8—C7—N2	118.6 (3)	O3—C3—C4	128.3 (3)
C12—C7—N2	121.1 (3)	N2—C3—C4	108.2 (3)
O14—C14—C4	118.5 (3)	C11—C12—C7	119.6 (4)
O14—C14—C15	117.6 (3)	C11—C12—H12	120.2
C4—C14—C15	123.9 (3)	C7—C12—H12	120.2
C9—C8—C7	118.6 (3)	C5—C6—H6A	109.5
C9—C8—H8	120.7	C5—C6—H6B	109.5
C7—C8—H8	120.7	H6A—C6—H6B	109.5
C16—C15—C20	118.8 (3)	C5—C6—H6C	109.5
C16—C15—C14	120.4 (3)	H6A—C6—H6C	109.5
C20—C15—C14	120.8 (3)	H6B—C6—H6C	109.5
C3—C4—C14	119.5 (3)	C11—C10—C9	118.5 (3)
C3—C4—C5	103.9 (3)	C11—C10—H10	120.7
C14—C4—C5	136.4 (3)	C9—C10—H10	120.7
N1—C5—C4	111.3 (3)	C18—C17—C16	121.7 (4)
N1—C5—C6	118.3 (3)	C18—C17—H17	119.2
C4—C5—C6	130.2 (3)	C16—C17—H17	119.2
C17—C18—C19	118.1 (4)	C18—C19—C20	120.5 (4)
C17—C18—C21	121.9 (4)	C18—C19—H19	119.7
C19—C18—C21	120.0 (4)	C20—C19—H19	119.7
C15—C16—C17	120.1 (4)	C18—C21—H21A	109.5
C15—C16—H16	119.9	C18—C21—H21B	109.5
C17—C16—H16	119.9	H21A—C21—H21B	109.5
C8—C9—C10	121.7 (4)	C18—C21—H21C	109.5
C8—C9—Cl1	118.7 (3)	H21A—C21—H21C	109.5
C10—C9—Cl1	119.5 (3)	H21B—C21—H21C	109.5
C10—C11—C12	121.2 (4)		
			
C3—N2—N1—C5	1.8 (4)	C7—C8—C9—C10	−1.0 (6)
C7—N2—N1—C5	174.8 (3)	C7—C8—C9—Cl1	177.9 (3)
C3—N2—C7—C8	158.0 (4)	C16—C15—C20—C19	−1.4 (5)
N1—N2—C7—C8	−13.5 (5)	C14—C15—C20—C19	−179.0 (3)
C3—N2—C7—C12	−21.7 (5)	N1—N2—C3—O3	177.2 (3)
N1—N2—C7—C12	166.8 (3)	C7—N2—C3—O3	5.1 (6)
C12—C7—C8—C9	1.5 (5)	N1—N2—C3—C4	−2.2 (4)
N2—C7—C8—C9	−178.3 (3)	C7—N2—C3—C4	−174.3 (3)
O14—C14—C15—C16	−41.9 (5)	C14—C4—C3—O3	−2.2 (6)
C4—C14—C15—C16	138.7 (4)	C5—C4—C3—O3	−177.7 (4)
O14—C14—C15—C20	135.7 (4)	C14—C4—C3—N2	177.1 (3)
C4—C14—C15—C20	−43.8 (5)	C5—C4—C3—N2	1.7 (4)
O14—C14—C4—C3	−6.5 (5)	C10—C11—C12—C7	1.8 (6)
C15—C14—C4—C3	173.0 (3)	C8—C7—C12—C11	−1.8 (5)
O14—C14—C4—C5	167.1 (4)	N2—C7—C12—C11	177.9 (3)
C15—C14—C4—C5	−13.5 (7)	C12—C11—C10—C9	−1.3 (6)
N2—N1—C5—C4	−0.7 (4)	C8—C9—C10—C11	1.0 (6)
N2—N1—C5—C6	−177.1 (3)	Cl1—C9—C10—C11	−178.0 (3)
C3—C4—C5—N1	−0.6 (4)	C19—C18—C17—C16	0.3 (6)
C14—C4—C5—N1	−174.9 (4)	C21—C18—C17—C16	−179.1 (3)
C3—C4—C5—C6	175.3 (4)	C15—C16—C17—C18	−1.7 (5)
C14—C4—C5—C6	1.0 (7)	C17—C18—C19—C20	0.5 (5)
C20—C15—C16—C17	2.2 (5)	C21—C18—C19—C20	179.9 (3)
C14—C15—C16—C17	179.8 (3)	C15—C20—C19—C18	0.1 (5)

### Hydrogen-bond geometry (Å, º)

**Table:**

*D*—H···*A*	*D*—H	H···*A*	*D*···*A*	*D*—H···*A*
O3—H3···O14	0.82	1.90	2.581 (3)	140
