# Supplementary figures and images for: Crystal structure of [1-(3-chloro­phen­yl)-5-hy­droxy-3-methyl-1H-pyrazol-4-yl](p-tol­yl)methanone
Source: Acta Crystallogr E Crystallogr Commun. 2015 Apr 2;71(Pt 5):o280–1. doi: 10.1107/S2056989015006258 (PMC4420059; doi:10.1107/S2056989015006258)

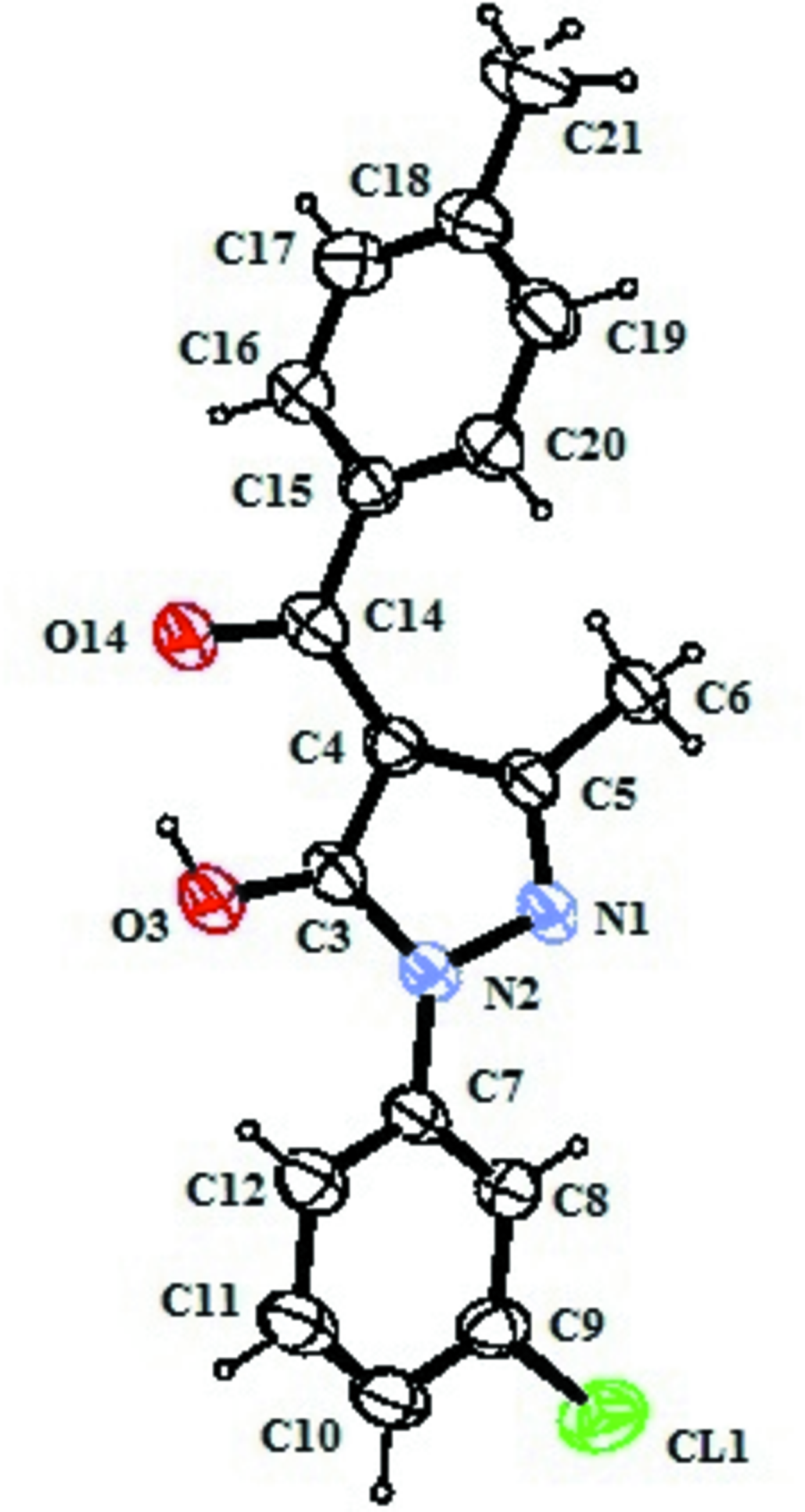

Supplement: Supplementary file 4 [file e-71-0o280-fig1.tif]

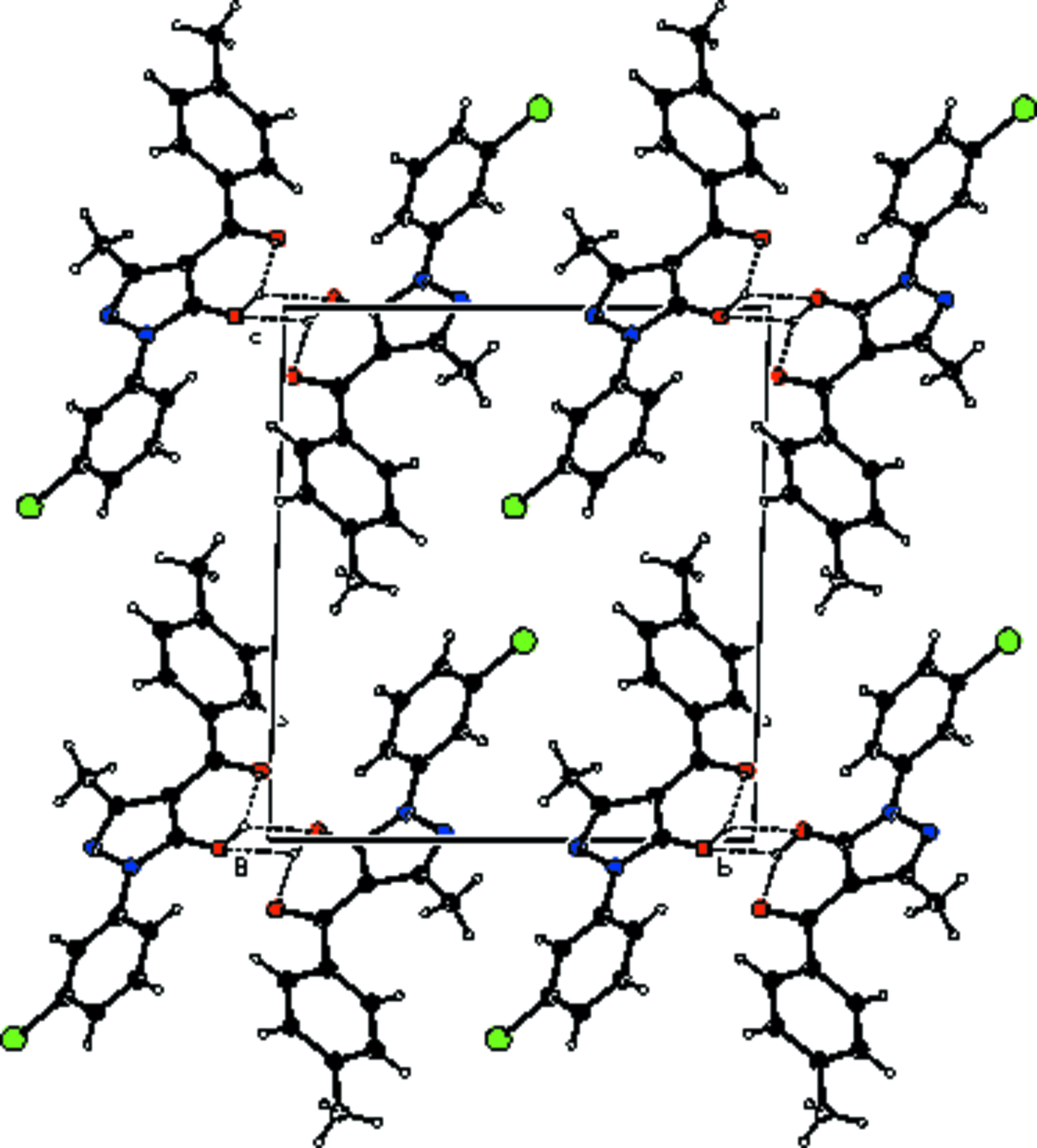

Supplement: Supplementary file 5 [file e-71-0o280-fig2.tif]
